# Human antibodies against *Mycobacterium avium* ssp. *paratuberculosis* combined with cytokine levels for the diagnosis and selection of Crohn’s disease patients for anti-mycobacterial therapy—A pilot study

**DOI:** 10.1371/journal.pone.0308911

**Published:** 2024-10-04

**Authors:** J. Todd Kuenstner, Peilin Zhang, Raghava Potula, Jean-Michel Galarneau, Horacio Bach

**Affiliations:** 1 Biology Department, Abilene Christian University, Abilene, Texas, United States of America; 2 PZM Diagnostics, Charleston, West Virginia, United States of America; 3 Lewis Katz School of Medicine, Temple University, Philadelphia, Pennsylvania, United States of America; 4 Sport Injury Prevention Research Centre, University of Calgary, Calgary, Alberta, Canada; 5 Division of Infectious Diseases, Department of Medicine, University of British Columbia, Vancouver, British Columbia, Canada; University of Illinois College of Veterinary Medicine, UNITED STATES OF AMERICA

## Abstract

Increasing evidence links a worldwide bacterial infection of cattle and other animal species by *Mycobacterium avium* ssp. *paratuberculosis* (MAP) to Crohn’s disease (CD). A large, FDA phase 2/3 controlled clinical trial of combination antimycobacterial antibiotic therapy for CD has been completed, and the report describing the trial is pending publication. The identification of MAP infection in CD patients will become increasingly important. Thus, it is desirable to develop MAP-based tests that accurately predict which CD patients have a MAP infection. A prospective, case-control laboratory test study of 199 subjects (61 CD patients and 138 non-CD controls) was performed using a panel of MAP antigens, including Hsp65, PknG, PtpA, CL1, and MAP IDEXX, which were measured under blind conditions in the plasma of the 199 subjects. Results showed that compared to any individual MAP antigen, combinations of antigens showed improved CD classification performance. For the Hsp65 antigen, the sensitivity (SEN), specificity (SPE), positive predictive value (PPV), negative predictive value (NPV), correct classification (CC), and area under the curve (AUC) were 59.02%, 58.70%, 38.71%, 76.42%, 59.3% and 0.606, respectively. For the best combination of MAP antibodies (Hsp65 and PknG), the SEN, SPE, PPV, NPV, CC, and AUC were 59.02%, 60.87%, 40.00%, 77.06%, 60.30%, and 0.631, respectively. Further improvement of the CD classification performance was achieved by combining IFN-γ, IL-8, and IL-17 cytokines with antibodies against MAP antigens, yielding SEN, SPE, PPV, NPV, CC, and AUC of 62.3%, 62.32%, 42.22%, 78.9%, 62.31% and 0.708, respectively. Thus, combinations of antibodies against MAP antigens and cytokine levels yield better CD diagnostic predictive performance than any individual antibodies against MAP antigens.

## Introduction

A recent review article on Crohn’s disease (CD) from the *Lancet* states that the cause and pathophysiology of CD are a result of the interplay between genetic susceptibility, environmental factors, and intestinal microflora, resulting in an abnormal mucosal immune response and compromised epithelial barrier function [1]. However, many researchers in this field have concluded that most patients with CD suffer from a chronic infection due to *Mycobacterium avium* ssp. *paratuberculosis* (MAP) [2].

MAP infection is the cause of Johne’s disease (JD), which results in chronic wasting, diarrhea, and cachexia in dairy and beef cattle and chronic wasting in sheep and goats. Moreover, MAP is widespread in the environment and causes disease in many wild animal species, including ruminants and rabbits. It has also been shown to cause disease in primates [3]. It has also been shown that MAP survives the pasteurization of milk [4, 5] and is present in the food chain, including in baby formula [6].

**Fig 1:**
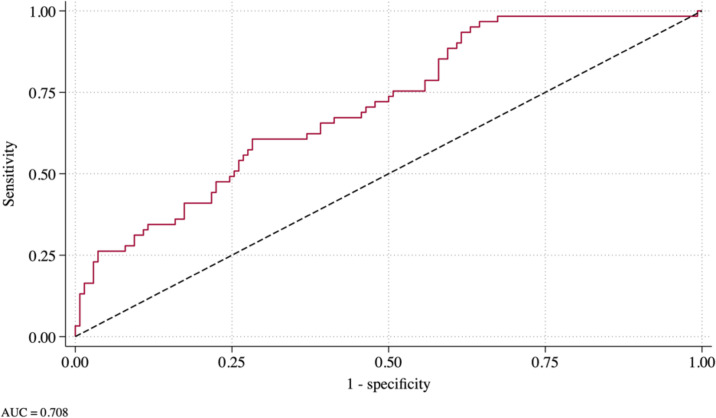
Analysis of the area under the curve. The area under the receiver operating characteristic curve (AUC) derived from the final, enhanced multivariable logistic regression is shown in the bottom panel of Table 4.

In an international multi-laboratory, prospective, case-control study (Temple University, TU), MAP was cultured from human blood samples using three direct culture methods and detected by a MAP phage assay in most study subjects [7]. In this study, viable MAP was cultured from the blood of the majority of the 201 subjects in the study (157 positives and 44 negatives, Fig 1), including most of the 61 CD patients and most of the 140 non-CD controls. The control arm of this study did not consist of healthy age-matched controls and instead contained many subjects with auto-immune diseases, including multiple sclerosis (MS), type 1 diabetes mellitus (T1DM), and rheumatoid arthritis (RA) that have been linked to MAP in epidemiologic studies [8–12].

Recently, RedHill Biopharma (Tel Aviv, Israel) reported the results of their FDA phase 2/3 clinical trial of anti-mycobacterial therapy (RHB-104) for treating CD patients with moderate to severe disease [13–15]. In addition, case series reports of the treatment of CD with anti-MAP therapy have described long-term functional cure/profound remission [16, 17].

Before treating CD patients with long-term anti-mycobacterial therapy, it is desirable to demonstrate that the patient has a MAP infection. In conjunction with the direct culture and indirect culture methods (MAP phage assay), the presence of antibodies against MAP antigens reported in this study bolsters the evidence of infection in CD patients and may be used to select CD patients for anti-mycobacterial therapy.

Several laboratories that participated in the international study of laboratory tests for MAP in CD and non-CD control subjects [7] determined the antibodies against MAP-related antigens in the population that was sampled. These antibodies against MAP antigens included the heat shock protein 65 (Hsp65), protein kinase G (PknG), protein phosphatase A (PtpA), MAP_1680 (CL1), and IDEXX MAP.

The rationale for choosing the MAP antigens was based on the fact that the *hsp65* gene amplification has been widely used to classify mycobacterial species in various clinical conditions, such as CD and Sjogren’s disease [18]. PtpA and PknG are two proteins secreted by MAP into macrophages upon infection and are necessary for the bacterium’s survival in the macrophage’s harsh environment [19, 20]. IgGs against both antigens were reported in different inflammatory conditions in humans [8–12]. CL1 is a cutinase-like protein expected to be secreted from MAP into the host [21], whereas MAP IDEXX is a MAP antibody test kit (IDEXX Laboratories, Inc., Westbrook, Maine, US) used for the detection of MAP in animals [22].

The single best antibody against MAP antigens for the diagnosis of CD in the TU study was Hsp65. In this study sample, a cutoff for positivity in CD cases was used based on an earlier study, which had one arm of CD patients and a control arm of healthy subjects [18]. Results of this study, the sensitivity (SEN) and specificity (SPE) of the anti-Hsp65 antibody were 90.4% and 74.8%, respectively. Here, combinations of the antibodies against MAP antigens were analyzed compared to the previously individually analyzed performance. The rationale behind the present study was to investigate whether the SEN, SPE, positive predictive value (PPV), negative predictive value (NPV), correct classification (CC), and area under the curve (AUC) of the antibodies against MAP antigens could be improved for better selection of CD patients for anti-MAP therapy like RHB-104.

## Materials and methods

This prospective case-control study included 199 subjects—61 patients with CD and 138 non-CD controls. The non-CD control group included 17 subjects with thyroid disease, not otherwise specified (THY), ten subjects with arthritis, not otherwise specified (ART), nine subjects with UC, five subjects with psoriasis (PSO), four subjects with type 1 diabetes mellitus (T1DM), two subjects with either type 2 diabetes mellitus (T2DM) or rosacea or irritable bowel syndrome (IBS), one subject with either asthma (AST) or multiple sclerosis (MS) or celiac disease (CEL) or eczema or lymphoma and 15 subjects with various combinations of UC, THY, ART, systemic lupus erythematosus (SLE), neurologic disease (NEU), AST, and CEL (Table 1).

**Table 1:** Health conditions in the non-CD controls.

Health conditions	N	Subject IDs
No reported disease*	69	5001, 5012, 5014, 5017, 5019, 5021, 5036, 5037, 5038, 5041, 5046, 5047, 5049, 5051, 5052, 5055, 5057, 5059, 5060, 5061, 5062, 5063, 5067, 5075, 5076, 5082, 5084, 5087, 5088, 5095, 5097, 5098, 5102, 5104, 5105, 5110, 5111, 5118, 5120, 5132, 5134, 5135, 5136, 5137, 5139, 5142, 5143, 5146, 5151, 5153, 5155, 5156, 5157, 5158, 5159, 5160, 5161, 5162, 5165, 5167, 5175, 5176, 5180, 5181, 5182, 5187, 5188, 5194, 5199, 5022, 5028, 5029, 5068, 5089, 5090, 5112, 5116, 5127, 5138, 5140, 5147, 5149, 5170, 5177, 5178, 5183
THY, not further specified	17	5022, 5028, 5029, 5068, 5089, 5090, 5112, 5116, 5127, 5138, 5140, 5147, 5149, 5170, 5177, 5178, 5183
ART, not further specified	10	5044, 5070, 5072, 5073, 5080, 5085, 5091, 5108, 5130, 5152
UC	9	5003, 5013, 5040, 5045, 5101, 5103, 5117, 5131, 5171
PSO	5	5024, 5119, 5122, 5158, 5191
T1DM	4	5025, 5042, 5077, 5184
T2DM	2	5168, 5185
Rosacea	2	5197, 5201
IBS	2	5107, 5172
AST	1	5083
MS	1	5164
Eczema	1	5179
CEL	1	5169
Lymphoma	1	5144
Combinations of UC, THY, ART, PSO, SLE, NEU, AST, and CEL	15	5004, 5015, 5031, 5043, 5054, 5069, 5092, 5113, 5114, 5125, 5128, 5173, 5189, 5193, 5200
All subjects in the non-CD control group	140	

*The study subjects were asked whether they had autoimmune diseases but not whether they had HTN, cardiovascular or peripheral vascular disease, or cancer. ART, arthritis; AST, asthma; CEL, celiac disease; IBS, irritable bowel syndrome; MS, multiple sclerosis; NEU, neurologic disease; PSO, psoriasis; SLE, systemic lupus erythematosus; THY, thyroid disease; T1DM, type 1 diabetes mellitus; T2DM, type 2 diabetes mellitus; UC, ulcerative colitis.

The study protocol was reviewed on 20 October 2017 by the TU IRB (IRB protocol # 24790). Subjects provided verbal consent. Further details on the study design and participants, diagnosis and diagnostic categorization, and the procedures have been described previously [7, 23]. In the current study, the collected data were previously analyzed between August 10, 2023, and January 15, 2024. The subject information was codified with no personal identification.

To examine the association between each antibody and its classification capability in terms of correctly predicting CD cases, means and medians were first compared by cohort with both a t-test and a non-parametric Wilcoxon rank-sum test, making no assumptions about the data. Second, a bivariate analysis using logistic regression was undertaken to assess the association between the antibodies and the outcome and also to compute classification statistics (S1 Fig). To achieve this, logistic regression was fit to predict the outcome of CD in a complete-case analysis, using the continuous form of the variable representing the antibody and its binary analog using prescribed cut-offs either derived from these data (through methods described below) or from previously published studies. The intersection of the highest sensitivity and specificity relative to the predicted probability of being a case generated by the logistic regression was then identified to find the probability cut-off at which classification statistics could be computed. SEN, SPE, PPV, NPV, and the proportion of observations correctly identified along with the AUC were generated from the logistic regression. Odds ratios and p-values were also generated and presented.

Subsequently, a multivariable model was constructed from the best combination of two antibodies to test whether classification statistics could be improved. This process was iterative and multiple logistic regressions were fit. The same approach was undertaken as in the bivariate analysis to compute classification statistics derived from the multivariable logistic regression.

Finally, to further improve on the model, the combination of the two most predictive antibodies was entered into a backward stepwise regression (p-value for entry = 0.05, p-value for removal = 0.1), locking these terms in the model and introducing a panel of 14 cytokines (S1 Fig). A final multivariable model was derived to compute classification statistics using the same approach discussed above, ignoring potential variance inflation issues and shrinkage. These were then compared to the classification statistics of the multivariable model of the two best antibodies. All work was performed in Stata (StataCorp. 2023. Stata Statistical Software: Release 18. College Station, TX: StataCorp LLC).

## Results

Of the 201 participants eligible for this analysis, 199 had data on all five antibodies and were retained for final analysis, resulting in 61 CD cases and 138 controls. Table 2 describes the sample of antibodies measured in the 199 participants stratified by disease status (CD vs. non-CD).

**Table 2:** Description of the 199 samples with complete information.

Antigen/Test		CD positive	CD negative	P-value
**Hsp65**	Mean (SD)	0.823 (0.290)	0.724 (0.265)	0.020
	Median (p25-p75)	0.797 (0.601–0.977)	0.665 (0.530–0.879)	0.017
**MAP IDEXX**	Mean (SD)	0.649 (0.404)	0.604 (0.382)	0.451
	Median (p25-p75)	0.641 (0.463–0.773)	0.620 (0.441–0.821)	0.754
**PknG**	Mean (SD)	0.218 (0.097)	0.253 (0.108)	0.038
	Median (p25-p75)	0.213 (0.153–0.284)	0.232 (0.178–0.298)	0.068
**PtpA**	Mean (SD)	0.063 (0.098)	0.082 (0.077)	0.147
	Median (p25-p75)	0.090 (0.24–0.125)	0.108 0.059–0.132)	0.222
**CL1**	Mean (SD)	0.090 (0.126)	0.117 (0.101)	0.101
	Median (p25-p75)	0.115 (-0.012–0.188)	0.152 (0.041–0.192)	0.282

Of the five antibodies tested, only Hsp65 and PknG showed differences between CD patients and non-CD controls, with Hsp65 showing the greatest differences where CD patients had significantly higher levels of the antibody (p = 0.020) following a t-test and a Wilcoxon non-parametric rank-sum test. For PknG, this relationship was reversed where CD patients had significantly lower levels of this antibody (p<0.038) following a t-test and borderline significantly lower following a non-parametric rank-sum test. CD patients also had slightly higher levels of MAP IDEXX, but this difference was not significantly greater than zero, while both PtpA and CL1 were lower in CD patients than in non-CD controls, a mean difference not significantly greater than zero.

Table 3 shows the classification statistics of each antibody in both continuous and binary forms following the published cut-off values discussed earlier and those derived from the data where applicable. The highest AUC was calculated for Hsp65 (AUC_continuous_ = 0.606) and PknG (AUC_continuous_ = 0.581), corresponding largely with the results shown in Table 2. Both showed significant associations between their continuous form and CD with Hsp65 producing, for each unit increase in the antibody levels, a 255% increase in the odds of diagnosis (p = 0.022), while this relationship was reversed for PknG where a 1 unit increase in antibody levels resulted in a 96% reduction in the odds of diagnosis (p = 0.038). For MAP IDEXX, PtpA, and CL1, the binary form of the antibodies did not perform well, with AUCs slightly improving for CL1 and PtpA with their continuous analogs, albeit never producing significant results in logistic regressions. The highest sensitivity was again computed for Hsp65, while the lowest sensitivity was found in both the categorical forms of IDEXX and CL1, resulting in specificities of 100% at those cut-off values.

**Table 3:** Bivariate analysis with classification statistics for each variable (N = 199).

		OR	P-value	SEN (%)	SPE (%)	PPV	NPV	CC (%)	AUC
**Hsp65**	Binary (>0.740)	2.159	0.014	57.380	61.590	39.770	76.580	60.300	0.595
	Continuous	3.549	0.022	59.020	58.700	38.710	76.420	59.300	0.606
**MAP IDEXX**	Binary (>0.550)	1.202	0.572	0.000	100.000	-	69.350	69.350	0.521
	Continuous	1.354	0.449	50.820	51.450	31.630	70.300	50.750	0.514
**PknG**	Binary (>0.135)	0.513	0.126	18.030	89.860	44.000	71.260	67.840	0.539
	Continuous	0.038	0.038	55.740	52.900	34.340	73.000	53.770	0.581
**PtpA**	Binary (>0.108)	0.743	0.337	57.380	50.000	33.650	72.630	52.260	0.537
	Binary (>0.126)	0.771	0.460	75.410	29.710	32.170	73.210	43.720	0.526
	Continuous	0.078	0.149	54.100	55.070	34.740	73.080	54.770	0.555
**CL1**	Binary (> = 0.151)	0.722	0.292	0.000	100.000	-	69.350	69.350	0.541
	Continuous	0.106	0.103	54.100	55.070	34.740	73.080	54.770	0.548

OR, odd ratio; SEN, sensitivity; SPE, specificity; PPV, positive predictive value; NPV, negative predictive value;

CC, correct classification.

The best combination of two antibodies based on the highest AUC produced by a multivariable logistic regression is shown in the top panel of Table 4. The OR for PknG and Hsp65 remain very similar to those shown in the bivariate table, with their statistical significance (p = 0.021, p = 0.057, respectively) remaining similar where PknG became borderline significantly related to diagnosis. The SEN of the logistic regression was 59.02% with a specificity of 60.87% and an AUC of 0.631, approximately 1% and 5%, higher than the AUC produced by the antibodies independently, respectively (Table 2). The enhanced model, including the two best cytokines from a stepwise regression forcing in the two best antibodies, is shown in the bottom panel of Table 4. The ORs for both Hsp65 and PknG are similar to what is shown in the top panel, with their levels of significance increasing (p = 0.006, p = 0.039, respectively). The SEN of the model increased to 62.30%, and the SPE of the model decreased to 62.32%, while the AUC increased by more than 0.07 to 0.708 (Fig 1). All three cytokines showed increases in the odds of diagnosis with an increase in the cytokine level with IFN-γ and IL-17α significant at p<0.05, while IL-8 had a p-value of 0.050.

**Table 4:** Results of multivariable logistic regression with highest AUC combining best two antibodies.

	OR	P-value	SEN (%)	SPE (%)	PPV	NPV	CC (%)	AUC
**Hsp65 Binary (>0.740)**	2.077	0.021	59.02	60.87	40.00	77.06	60.30	0.631
**PknG Continuous**	0.043	0.057						
**Intercept**	0.652	0.331						
**Hsp65**	2.508	0.006	62.3	62.32	42.22	78.9	62.31	0.708
**PknG**	0.025	0.039						
**IFN-γ**	1.541	0.013						
**IL-8**	1.013	0.050						
**IL-17α**	1.652	0.025						
**Intercept**	0.206	0.005						

OR, odd ratio; SEN, sensitivity; SPE, specificity; PPV, positive predictive value; NPV, negative predictive value;

CC, correct classification.

## Discussion

The results of this study are likely those of earlier studies and add supportive evidence that MAP bacteremia is found in the human population in health and disease and that is probably an important cause of CD.

It was reported in a study analyzing the blood of 246 IBD patients and 100 healthy controls that showed that 16% and 47% had MAP-positive blood samples by nested PCR, respectively [24]. Likewise, a study of CD (n = 173), ulcerative colitis (n = 105) patients, and healthy controls (n = 188) found that 21.38%, 19.04%, and 45.2%, respectively, had MAP-positive blood samples by PCR [25]. These two studies found that more controls than CD patients were positive for MAP (64% of the non-CD controls versus 57% of the CD patients grew MAP by the Pozzato culture method) [7].

However, we used culture methods which demonstrated that the MAP cells in the blood samples were viable, while the other two studies used PCR that cannot differentiate MAP DNA from viable and dead bacteria. In addition, in our study, we examined a small group of healthy and diseased subjects and showed that most have a persistent infection rather than a transient bacteremia. It will be important to confirm the finding of persistent infection in a much larger cohort. Moreover, the two studies referred to above, posited that the smaller percentage of MAP-positive blood samples in the IBD group compared to the healthy controls was most likely due to the anti-MAP activity of the therapy the patients received and we agree with this opinion. All CD patients in our study were receiving treatment which in most cases was either infliximab or adalimumab.

Our findings also resemble those obtained in another study using MAP phage positivity in a cattle population which included animals with JD. In this study, MAP bacteremia was noted in asymptomatic and subclinical cattle without JD. By one of the more sensitive assays in their study, phage/qPCR, 72% (23/32) of JD and 35% (6/17) of controls were MAP positive. Interestingly, with fecal PCR 75% (24/32) of JD and none (0/17) of the healthy controls were MAP positive [26].

In conjunction with the direct culture and indirect culture methods (MAP phage assay), the antibodies against MAP antigens reported in this study bolster the evidence of infection in CD patients and may be used to select CD patients for anti-mycobacterial therapy.

This statistical analysis of five antibodies determined in the 199 TU study subjects shows that a combination of antibodies against MAP antigens improves the SEN, SPE, PPV, NPV, and CC for CD diagnosis in comparison to any single MAP antibody. Further improvement in all these values was achieved using a combination of the antibodies and three cytokines, typically elevated in CD, i.e., IL-8, IL-17α, and IFN-γ [27].

If these antibodies against MAP antigen tests are applied to a large sample with robust, healthy, and age-matched controls, the starting point is using a single antibody, such as against the Hsp65 antigen, which showed SEN and SPE of 90.4% and 74.8%, respectively, in a sample using healthy Red Cross donors as controls [18]. When tested in large samples with robust controls, incremental improvements of SEN, SPE, PPV, NPV, and CC value using combinations of the antibodies should yield excellent CD prediction and/or corroboration of the disease suspected by the clinician.

We anticipate that antibodies against MAP antigen testing will be improved as new antibodies are discovered and that it will be possible to diagnose and treat patients for CD with an approach that combines a clinical history of typical signs and symptoms with these laboratory tests. These tests should ameliorate the difficulty in some cases of initially establishing the diagnosis of CD and reduce the use of invasive procedures required for CD diagnosis, such as endoscopy and biopsy.

Although the data from this sample population, on the surface, may not appear compelling, significantly improved test parameters are anticipated in future studies, which will include much larger numbers of healthy and age-matched controls. Notably, the sample population in the TU study was suboptimal because so many of the subjects in the control arm suffered from autoimmune diseases, including many that have been epidemiologically linked to MAP.

## Conclusions

The statistical analysis of five antibodies against MAP antigens determined in the 199 TU study subjects shows that a combination of them improves the SEN, SPE, PPV, NPV, and CC values for CD diagnosis in comparison to any single antibody. Further improvement in all these values was achieved using a combination of antibodies and three cytokines, typically elevated in CD, i.e., IL-8, IL-17α, and IFN-γ. These antibodies should prove useful for the selection of CD patients for anti-mycobacterial therapy.

## Supporting information

S1 Fig MAP-Crohn’s data. (XLSX)
